# Colchicine and diabetes in patients with chronic coronary artery disease: insights from the LoDoCo2 randomized controlled trial

**DOI:** 10.3389/fcvm.2023.1244529

**Published:** 2023-10-06

**Authors:** Niekbachsh Mohammadnia, Jan Los, Tjerk S. J. Opstal, Aernoud T. L. Fiolet, John W. Eikelboom, Arend Mosterd, Stefan M. Nidorf, Charley A. Budgeon, Jan G. P. Tijssen, Peter L. Thompson, Cees J. Tack, Suat Simsek, Willem A. Bax, Jan H. Cornel, Saloua El Messaoudi

**Affiliations:** ^1^ Department of Cardiology, Radboudumc, Nijmegen, Netherlands; ^2^ Department of Cardiology, Northwest Clinics, Alkmaar, Netherlands; ^3^ Department of Cardiology, University Medical Center Utrecht, Utrecht, Netherlands; ^4^ Dutch Network for Cardiovascular Research (WCN), Utrecht, Netherlands; ^5^ Department of Medicine, McMaster University, Hamilton, ON, Canada; ^6^ Department of Cardiology, Meander Medical Center, Amersfoort, Netherlands; ^7^ Heart and Vascular Research Institute of Western Australia, Perth, WA, Australia; ^8^ GenesisCare Western Australia, Perth, WA, Australia; ^9^ School of Medicine, University of Western Australia, Perth, WA, Australia; ^10^ Department of Cardiology, Amsterdam University Medical Centers, Amsterdam, Netherlands; ^11^ Cardialysis BV, Rotterdam, Netherlands; ^12^ Sir Charles Gairdner Hospital, Perth, WA, Australia; ^13^ Department of Internal Medicine, Radboudumc, Nijmegen, Netherlands; ^14^ Department of Internal Medicine, Northwest Clinics, Alkmaar, Netherlands; ^15^ Department of Internal Medicine, Amsterdam University Medical Centers, Amsterdam, Netherlands

**Keywords:** diabetes mellitus, coronary artery disease, colchicine, inflammation, prevention

## Abstract

**Introduction:**

Despite optimal treatment, patients with chronic coronary artery disease (CAD) and diabetes mellitus (DM) are at high risk of cardiovascular events, emphasizing the need for new treatment options. The Low-Dose Colchicine 2 (LoDoCo2) trial demonstrated that colchicine reduces cardiovascular risk in patients with chronic CAD. This analysis determines the efficacy of colchicine in patients with chronic CAD and DM as well as the effect of colchicine on the development of new-onset type 2 diabetes mellitus (T2DM).

**Methods:**

The LoDoCo2 trial randomized 5,522 patients to placebo or colchicine 0.5 mg once daily, with a median follow-up of 28.6 months. The primary composite endpoint was cardiovascular death, spontaneous myocardial infarction, ischemic stroke, or ischemia-driven revascularization. The effect of its treatment in patients with and without DM was evaluated by including an interaction term in the model.

**Results:**

A total of 1,007 participants (18.2%) had T2DM at baseline. The adjusted hazard ratio (HR) [(95% confidence interval (CI)] for the primary endpoint in the T2DM group was 1.52 (1.15–2.01, *p *< 0.01) compared with the group without T2DM. The HR for the treatment effect on the primary endpoint was 0.87 (0.61–1.25) in participants with T2DM and 0.64 (0.51–0.80) in participants without diabetes (*p*_interaction _= 0.14). The incidence of new-onset T2DM was 1.5% (34 out of 2,270) in the colchicine group and 2.2% (49 out of 2,245) in the placebo group (*p* = 0.10).

**Discussion:**

In conclusion, based on the current evidence, the beneficial effects of colchicine on cardiovascular endpoints are consistent regardless of DM status. The potential benefits of colchicine in preventing new-onset DM need further investigation. These findings are only hypothesis-generating and require larger prospective trials to confirm the results.

## Introduction

Atherosclerosis is an inflammatory disease (1). Patients with type 2 diabetes mellitus (T2DM) are at increased risk of developing atherosclerotic cardiovascular disease (2). Patients with chronic coronary artery disease (CAD) who also have diabetes mellitus are particularly at high risk for recurrent cardiovascular events, which underscores the need for new therapeutic options (3).

T2DM is a multifactorial disease characterized by insulin resistance and relative insulin deficiency attributed to islet beta-cell failure causing hyperglycemia and dyslipidemia (4). Obesity is a low-grade chronic inflammatory disease and is the most crucial factor in the development of T2DM and related metabolic disorders (5, 6). Therefore, the role of inflammation in general and oligomerization of nucleotide-binding domain-, leucine-rich repeat-, and pyrin domain-containing protein 3 (NLRP3) inflammasome in particular on the onset and progression of T2DM has been hypothesized (7, 8). Small studies suggest that therapy that targets the inflammatory cytokine interleukin-1 (IL-1) can improve glycemic control in T2DM (9, 10). However, in the larger substudy of the Canakinumab Anti-Inflammatory Thrombosis Outcomes Study (CANTOS), targeting interleukin-1β (IL-1β) did not result in better glycemic control or a reduction in the incidence of new-onset T2DM (11).

The Colchicine Cardiovascular Outcomes Trial (COLCOT) and Low-Dose Colchicine 2 (LoDoCo2) trial demonstrated that low-dose colchicine reduces the risk for cardiovascular death, myocardial infarction, ischemic stroke, or ischemia-driven coronary revascularization in unselected patients with recent myocardial infarction and chronic CAD, respectively (12, 13). Colchicine has broad anti-inflammatory effects that include inhibition of the NLRP3 inflammasome and polymerization of tubulin that affects leukocyte function (14–16). In this study, we assessed the efficacy of colchicine on cardiovascular events and the effect of colchicine on the development of new-onset T2DM in patients with chronic CAD with and without diabetes mellitus (17).

## Methods

The LoDoCo2 trial (ACTRN12614000093684) was a double-blind randomized clinical trial, with a total of 5,522 patients randomized to colchicine 0.5 mg (*n* = 2,762) or placebo once daily (*n* = 2,760). Recruitment started in 13 centers in Western Australia in 2014 and was expanded to 30 centers in the Netherlands in 2016. Enrollment ended in 2018. The median follow-up time was 28.6 months (interquartile range, 20.5–44.4). The patients were eligible if they were aged 35–82 years, had established chronic CAD, were clinically stable for at least 6 months prior to randomization, and were able to tolerate colchicine during a 30-day run-in period. Randomization was performed in a double-blind 1:1 fashion to colchicine or placebo that was performed by a computerized algorithm. The primary efficacy endpoint was composed of major adverse cardiovascular events (MACE+), namely, cardiovascular death, spontaneous myocardial infarction, ischemic stroke, or ischemia-driven revascularization. Secondary endpoints consisted of the aforementioned events, separately. The endpoints were revised several times before unblinding of the data. All cardiovascular events were judged by a clinical events committee blinded to treatment allocation.

Calculation of the sample size for the original trial has previously been published and details can be found in the study protocol (12, 18). To summarize, with 5,447 randomized participants, the study would achieve a beta of <0.10 at a two-sided alpha of 0.05 to detect a difference of 30% in the incidence of the primary composite endpoint between treatment groups. Diabetes status and insulin treatment were assessed at the time of randomization. A participant who was not on diabetes treatment at the time of randomization and subsequently started treatment was defined as having “new-onset T2DM.” The trial protocol was approved by a centralized institutional review board in each participating country (MEC-U, Nieuwegein, Netherlands; and Sir Charles Gairdner Group HREC, Perth, Australia). All patients provided written informed consent. Additional details of the design, statistical analyses, baseline characteristics of the participants, and primary results of LoDoCo2 have been published (12, 18).

### Statistical analyses

The baseline characteristics stratified by diabetes status are shown as mean ± standard deviation for normally distributed variables, as median with the interquartile range if non-normally distributed, and as proportions with percentages. Normality was visually assessed using histograms and Q–Q plots. Differences between baseline characteristics were assessed with independent sample *t*-tests or chi-squared tests where applicable.

Cox proportional hazard models were used to investigate univariable relationships between DM status and endpoints in the placebo group. Multivariable adjustment was performed with baseline variable predictors of the primary outcome as previously reported by using the forward Wald method (19). The variables were as follows: age >70 years, current smoker, a history of both coronary artery bypass grafting and percutaneous coronary intervention, a combination of oral anticoagulants and dual antiplatelet therapy, and no statin use.

Treatment effects for primary and secondary efficacy endpoints were presented by diabetes status. Kaplan–Meier estimates were plotted by treatment group (colchicine or placebo) and diabetes status. The interactions between the treatment group and diabetes status were evaluated with the addition of treatment and the treatment-by-diabetes status variable interaction. The difference in the incidence of new-onset T2DM between treatment groups was calculated using Fisher's exact test because the time to new-onset T2DM was not registered in the LoDoCo2 trial.

Hazard ratio (HR) with 95% confidence intervals (CI) was calculated, and the calculated *p*-values were two-tailed.

## Results

### Baseline characteristics

A total of 1,007 (18.2%) of the 5,522 participants in the LoDoCo2 trial had T2DM at baseline (Table 1), of whom 287 (28.5%) used insulin. The participants with T2DM were slightly older; more frequently had a history of atrial fibrillation, hypertension, and impaired renal function (eGFR of <60 ml/min/1.73 m^2^); and had more often undergone coronary artery bypass grafting (CABG) compared with participants without T2DM. Renin angiotensin inhibitors, beta-blockers, and anticoagulants were used more frequently by participants with T2DM.

**Table 1:** Baseline characteristics of patients in LoDoCo2 stratified by DM status.

	Type 2 diabetes (*n* = 1,007)	No diabetes (*n* = 4,515)	*p*-value^a^
Demographics			
Age, mean (SD), years	66.7 (8.0)	65.6 (8.7)	<0.001
Female, No. (%)	144 (14.3)	702 (15.5)	0.32
Clinical characteristics			
Hypertension, No. (%)	628 (62.4)	2,180 (48.3)	<0.001
Current smoker, No. (%)	127 (12.7)	521 (11.6)	0.34
Insulin-dependent DM, No. (%)	287 (28.5)		
Creatinine clearance <60 ml/min/1.73 m^2^, No. (%)	76 (7.5)	230 (5.1)	<0.01
Prior acute coronary syndrome, No. (%)	843 (83.7)	3,815 (84.5)	0.54
Prior coronary revascularization, No. (%)	856 (85.0)	3,765 (83.4)	0.21
Coronary artery bypass grafting, No. (%)	189 (18.8)	521 (11.5)	<0.001
Percutaneous coronary intervention, No. (%)	743 (73.8)	3,434 (76.1)	0.13
History of atrial fibrillation, No. (%)	138 (13.7)	511 (11.3)	0.03
Medication at enrollment			
Single antiplatelet therapy, No. (%)	654 (64.9)	3,047 (67.5)	0.12
Dual antiplatelet therapy, No. (%)	234 (23.2)	1,046 (23.2)	0.96
Anticoagulant, No. (%)	144 (14.3)	528 (11.7)	0.02
No antiplatelet agent or anticoagulant, No. (%)	3 (0.3)	12 (0.3)	0.86
Statin, No. (%)	938 (93.1)	4,250 (94.1)	0.24
Renin angiotensin inhibitor, No. (%)	815 (80.9)	3,145 (69.7)	<0.001
Beta-blocker, No. (%)	695 (69.0)	2,732 (60.5)	<0.001

^a^ *p*-values for comparison between groups were calculated using the chi-square test for proportions and independent sample *t*-test for means.

### Endpoints in relation to T2DM status at baseline

**Figure 1:**
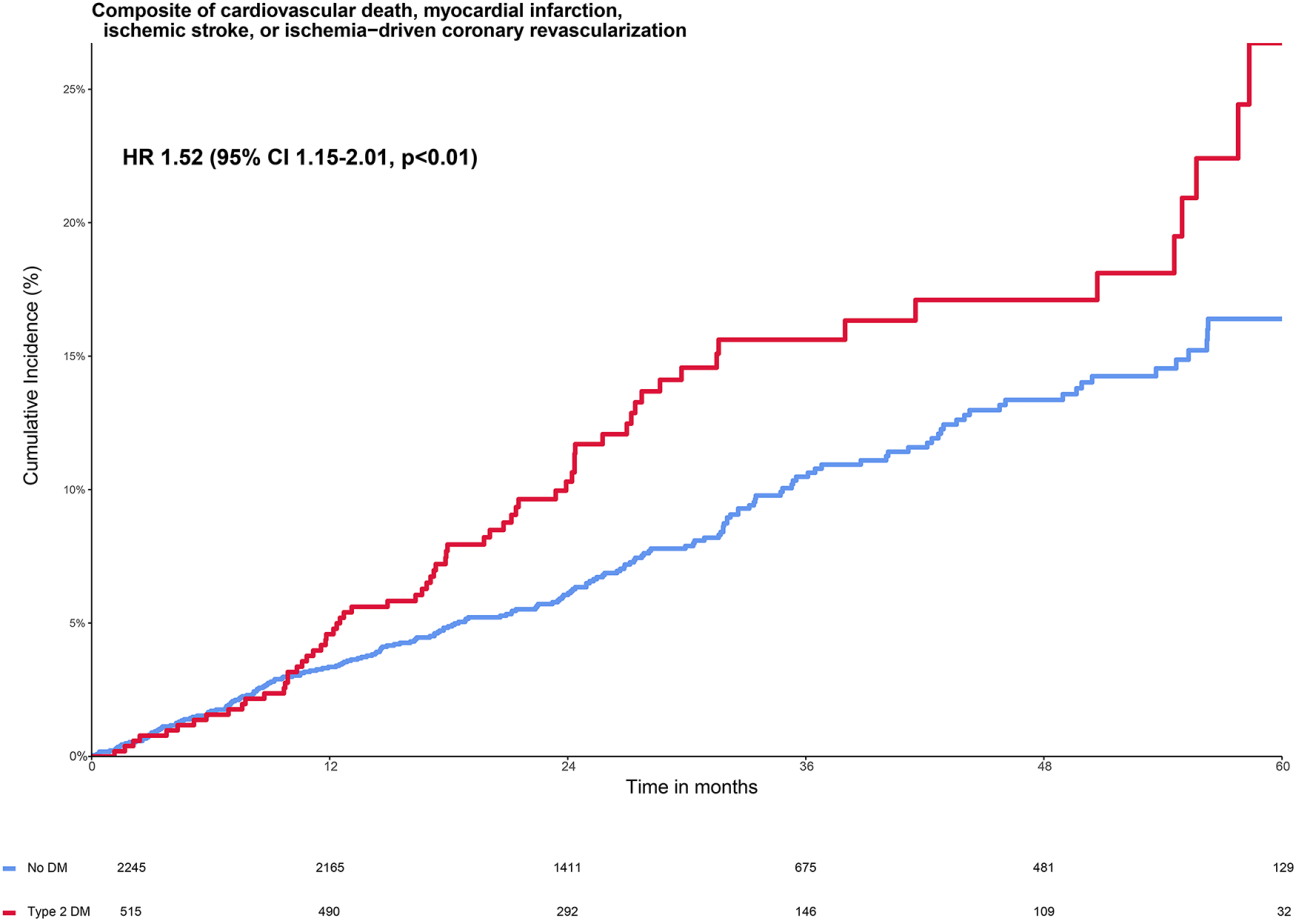
Cumulative incidence of primary composite endpoint stratified by diabetes status in the placebo group. The Kaplan–Meier curve shows the cumulative incidence of the primary composite endpoint of cardiovascular death, myocardial infarction, ischemic stroke, or ischemia-driven coronary revascularization in patients with type 2 diabetes (red line) and without type 2 diabetes (blue line). The figure shows the increased risk of patients with diabetes vs. without diabetes on the primary composite endpoint with a hazard ratio of 1.52 (95% 1.15–2.01, *p* < 0.01), adjusted for the baseline characteristics from Table 1 that were independent predictors of the primary endpoint: age >70 years, current smoker, a history of both coronary artery bypass grafting and percutaneous coronary intervention, a combination of oral anticoagulants and dual antiplatelet therapy, and no statin use (19).

The primary composite endpoint of cardiovascular death, spontaneous myocardial infarction, ischemic stroke, or ischemia-driven coronary revascularization (MACE+) in the placebo group occurred in 13.0% (67 out of 515) participants with T2DM and in 8.8% (197 out of 2,245) of the participants without diabetes (Figure 1 and Table 2). Adjusted HR (95% CI) for MACE+ in the T2DM group was 1.52 (95% CI 1.15–2.01, *p *< 0.01) compared with the group without diabetes (Figure 1). The participants with T2DM had an increased hazard for all secondary endpoints compared with the participants without diabetes, although this did not reach statistical significance for spontaneous myocardial infarction, cardiovascular death, and ischemic stroke (Table 2).

**Table 2:** Effect of diabetes status at baseline on the endpoints in the placebo group.

	Events	Unadjusted^a^		Adjusted for multiple variables^b^	
Subgroup	% (*n*/*N*)	HR (95% CI)	*p*-value	HR (95% CI)	*p*-value
Composite of cardiovascular death, spontaneous myocardial infarction, ischemic stroke, or ischemia-driven coronary revascularization					
No diabetes mellitus	8.8 (197 out of 2,245)				
Type 2 diabetes mellitus	13.0 (67 out of 515)	1.54 (1.16–2.03)	<0.01	1.52 (1.15–2.01)	<0.01
Composite of cardiovascular death, spontaneous myocardial infarction, or ischemic stroke					
No diabetes mellitus	5.2 (116 out of 2,245)				
Type 2 diabetes mellitus	8.0 (41 out of 515)	1.57 (1.10–2.24)	0.01	1.55 (1.08–2.21)	0.02
Spontaneous myocardial infarction					
No diabetes mellitus	3.9 (87 out of 2,245)				
Type 2 diabetes mellitus	5.6 (29 out of 515)	1.47 (0.96–2.24)	0.07	1.45 (0.95–2.20)	0.09
Ischemia-driven coronary revascularization					
No diabetes mellitus	5.8 (130 out of 2,245)				
Type 2 diabetes mellitus	9.1 (47 out of 515)	1.63 (1.17–2.28)	<0.01	1.63 (1.16–2.27)	<0.01
Cardiovascular death					
No diabetes mellitus	0.8 (17 out of 2,245)				
Type 2 diabetes mellitus	1.6 (8 out of 515)	2.03 (0.88–4.71)	0.10	2.06 (0.89–4.78)	0.09
Ischemic stroke					
No diabetes mellitus	0.8 (17 out of 2,245)				
Type 2 diabetes mellitus	1.4 (7 out of 515)	1.78 (0.74–4.30)	0.20	1.74 (0.72–4.21)	0.22

Analysis compared diabetes vs. no diabetes.

^a^ Hazard ratios adjusted for treatment were only marginally different compared with unadjusted hazard ratios.

^b^ Adjusted for the baseline characteristics from Table 1 that were independent predictors of the primary endpoint: age >70 years, current smoker, a history of both coronary artery bypass grafting and percutaneous coronary intervention, a combination of oral anticoagulants and dual antiplatelet therapy, and no statin use (19).

### Endpoints in relation to T2DM status and randomized treatment

**Figure 2:**
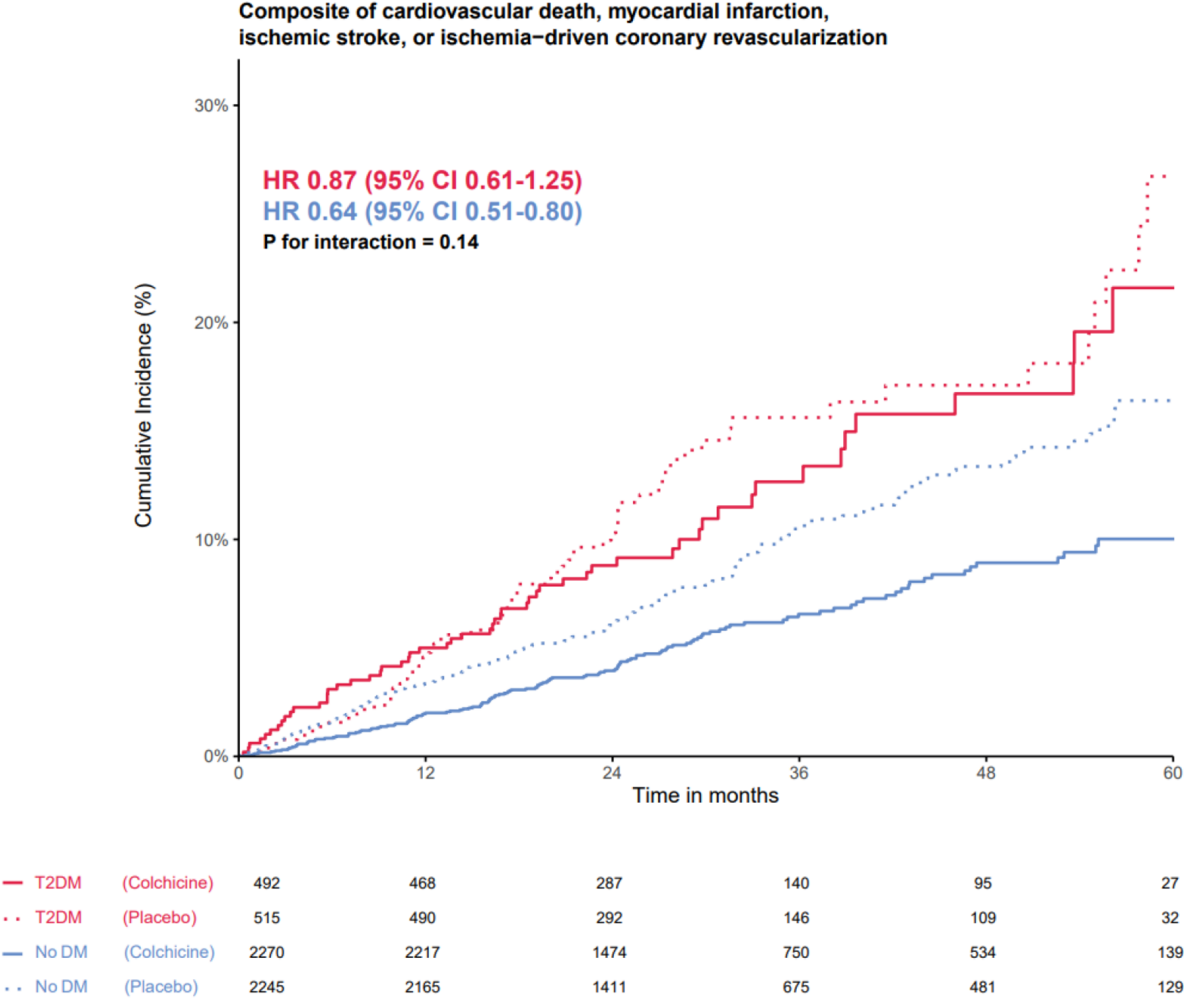
The efficacy of colchicine vs. placebo on the primary composite endpoint stratified by diabetes status. The Kaplan–Meier curve shows the effect of colchicine 0.5 mg (solid lines) once daily vs. placebo (dotted lines) on the primary composite endpoint of cardiovascular death, myocardial infarction, ischemic stroke, or ischemia-driven coronary revascularization in patients with type 2 diabetes (red lines) and without type 2 diabetes (blue lines). The hazard ratios for the treatment effect of colchicine did not show an interaction between the group with and without DM on the primary composite endpoint.

**Figure 3:**
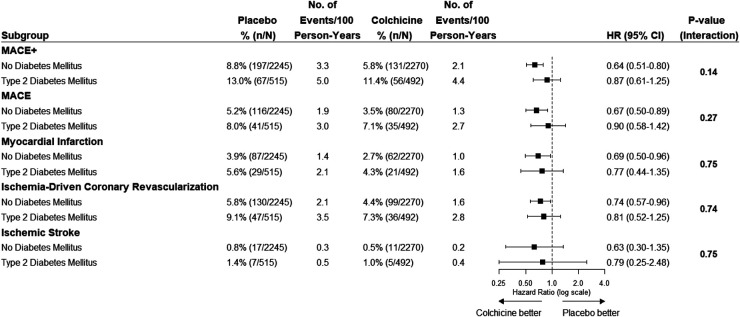
The efficacy of colchicine vs. placebo on the primary composite endpoint and secondary endpoints stratified by diabetes status. The figure shows the effect of colchicine 0.5 mg once daily vs. placebo on the primary composite endpoint of cardiovascular death, myocardial infarction, ischemic stroke, or ischemia-driven coronary revascularization and secondary outcomes. The hazard ratios for the treatment effect of colchicine did not show an interaction between the group with and without diabetes on the primary composite endpoint and secondary endpoints. MACE+, primary composite outcome of cardiovascular death, spontaneous myocardial infarction, ischemic stroke, or ischemia-driven coronary revascularization; MACE, cardiovascular death, spontaneous myocardial infarction, or ischemic stroke.

The effects of colchicine on the primary composite endpoint and, separately, MACE, spontaneous myocardial infarction, and ischemia-driven coronary revascularization were consistent in the participants with and without T2DM (Figures 2, 3). No DM status-by-treatment interaction was found (Figure 3). The cumulative incidence of myocardial infarction and ischemia-driven coronary revascularization in the colchicine and placebo groups in the participants with diabetes at baseline and without diabetes are shown in the Supplementary material (Supplementary Figures S1, S2). Although the colchicine-treated participants had more non-cardiovascular death compared to placebo, no significant difference was reported in any single cause of non-cardiovascular fatalities across treatment groups.

### New-onset T2DM

New-onset T2DM occurred in 83 participants during follow-up. No significant difference was found in the baseline characteristics between the colchicine and placebo groups (Table 3). The incidence of new-onset T2DM was lower in the colchicine treatment-arm group (1.5%, 34 out of 2,270) compared with the placebo group (2.2%, 49 out of 2,245). However, no statistically significant difference was reported (*p* = 0.10).

**Table 3:** Baseline characteristics by treatment group of participants that developed new-onset type 2 diabetes.

	Colchicine (*n* = 34)	Placebo (*n* = 49)	*p*-value^a^
Demographics			
Age, mean (SD), years	60.9 (8.6)	62.6 (9.8)	0.40
Female, No. (%)	3 (8.8)	10 (20.4)	0.15
Clinical characteristics			
Hypertension, No. (%)	18 (52.9)	22 (44.9)	0.47
Current smoker, No. (%)	10 (30.3)	11 (22.4)	0.42
Creatinine clearance <60 ml/min/1.73 m^2^, No. (%)	3 (8.8)	3 (6.1)	0.64
Prior acute coronary syndrome, No. (%)	30 (88.2)	43 (87.8)	0.95
Prior coronary revascularization, No. (%)	31 (91.2)	41 (83.7)	0.32
Coronary artery bypass grafting, No. (%)	2 (5.9)	7 (14.3)	0.23
Percutaneous coronary intervention, No. (%)	30 (88.2)	36 (73.5)	0.10
History of atrial fibrillation, No. (%)	2 (5.9)	5 (10.2)	0.49
Medication at enrollment			
Single antiplatelet therapy, No. (%)	20 (58.8)	26 (53.1)	0.60
Dual antiplatelet therapy, No. (%)	13 (38.2)	18 (36.7)	0.89
Anticoagulant, No. (%)	1 (2.9)	7 (14.3)	0.09
No antiplatelet agent or anticoagulant, No. (%)	0 (0)	0 (0)	—
Statin, No. (%)	33 (97.1)	49 (100)	0.23
Renin angiotensin inhibitor, No. (%)	27 (79.4)	37 (75.5)	0.68
Beta-blocker, No. (%)	24 (70.6)	35 (71.4)	0.93

^a^ *p*-values for comparison between groups were calculated using the chi-square test for proportions and independent sample *t*-test for means.

### Premature permanent discontinuation of study medication

Of the participants with T2DM, 16 (3.3%) participants reported experiencing side effects that resulted in premature permanent discontinuation of colchicine compared with 14 (2.7%) participants in the placebo group (Supplementary Table S1).

## Discussion

This LoDoCo2 substudy confirms that patients with chronic CAD who also have T2DM are at higher risk of MACE than patients without T2DM. It also demonstrates that colchicine produces consistent benefits in preventing recurrent MACE in patients with and without T2DM. While the incidence of new-onset T2DM in the colchicine group was numerically lower, this difference was not statistically significant.

The hypothesized underlying role of inflammation in T2DM relates to the belief that obesity results in the recruitment of macrophages into the adipose tissue and subsequent induction of a pro-inflammatory environment, which contributes to insulin resistance (20–22). Concomitant relative islet beta-cell dysfunction attributed to either inflammation or genetic predisposition results in insulin deficiency and further propels hyperglycemia contributing to the development or progression of T2DM (21–25). Therapy with IL-1 and IL-1β antibodies specifically targets these cytokines mediated by the NLRP3 inflammasome, whereas colchicine directly attenuates the inflammasome (26, 27). Also, the mechanisms of actions of colchicine reach beyond the IL-1 pathway (15, 28). Therefore, a wider therapeutic effect could be expected from colchicine, but we were unable to demonstrate this.

The effects of anti-inflammatory therapy on cardiovascular events had also been assessed in the pre-specified DM substudy of CANTOS. The study showed that the beneficial effect of the IL-1β inhibitor canakinumab in patients with a baseline high-sensitivity C-reactive protein of ≥2 mg/L and history of MI did not differ between participants with and without T2DM (11, 29). The current LoDoCo2 substudy adds to the accumulating evidence confirming the consistent reduction of the composite primary endpoint by the anti-inflammatory drug colchicine, independent of T2DM status. Because patients with DM are at higher risk of adverse cardiovascular events, they can be expected to derive greater absolute benefits from colchicine than patients without DM. This was also recently hypothesized in patients with type 1 diabetes (30).

The effects of anti-IL-1 therapy on glycemic control have also been studied in patients with T2DM. A study on the use of anti-IL-1 therapy in patients with rheumatoid arthritis and T2DM reported improved glycemic control (9). A clinical trial in 70 patients with an IL-1-receptor antagonist in T2DM improved glycemic control and reduced inflammation, although these findings have not been confirmed (10, 31). In CANTOS, the HbA1c values were not affected by IL-1β inhibition (11). This could not be explored in the LoDoCo2 study because glycemic control was not measured.

The effect of anti-inflammatories on the incidence of new-onset T2DM was only prospectively studied in CANTOS, showing no treatment difference between the treatment and placebo groups (11). For colchicine, two retrospective cohort studies compared new-onset T2DM in patients treated with colchicine or without colchicine for gout. Both studies showed a reduction in new-onset T2DM in the colchicine population, compared with the findings of the present study (32–34). The numerical lower rate of new-onset T2DM in the present study is consistent with the hypothesis that anti-inflammatory therapy by using colchicine possibly prevents or delays new-onset T2DM, although low numbers preclude any definitive conclusions. Statins also have anti-inflammatory properties, but meta-analyses have suggested an increase in the risk of new-onset T2DM with statins (34–36). This raises the possibility that different (inflammatory) pathways are involved in new-onset DM and the development of atherosclerosis.

Many currently available therapies for T2DM have anti-inflammatory properties, but it is not known whether their anti-inflammatory effects are beneficial (37). Several ongoing trials are investigating the role of anti-inflammatory therapy in patients with CAD and diabetes, such as the ZEUS trial with ziltivekimab (NCT05021835) and the CLEAR SYNERGY trial with colchicine (NCT03048825).

### Limitations

The present study contains several limitations. First, the incidence of new-onset DM could have been underestimated in the LoDoCo2 trial population because new-onset DM was defined at the start of pharmacological treatment, whereas non-pharmacological (lifestyle) recommendations may precede pharmacological treatment. Second, the LoDoCo2 trial was not designed or powered to assess the effect of colchicine in patients with DM or the incidence of new-onset DM. Third, changes in the treatment of DM and measures of glycemic control were not routinely collected. Information on other variables influencing inflammation was also unavailable, such as body mass index, diet, and physical activity (5, 6, 37, 38). Lastly, the effects of colchicine on any specific cause of death were previously analyzed, showing no association with any cause (39). Subgroup analyses by DM status cannot further inform the effects of colchicine vs. placebo on any specific cause of death.

In conclusion, based on the current evidence, the beneficial effects of colchicine on cardiovascular endpoints are consistent regardless of DM status. The potential benefits of colchicine in preventing new-onset DM require further investigation. These findings are only hypothesis-generating and require larger prospective trials to confirm the results.

## Data Availability

The datasets presented in this article are not readily available because individual participant data that underlie the results reported in this article, after de-identification (text, tables, figures, and supplements), will be made accessible for analyses approved by the LoDoCo2 steering committee. The data will be accessible for researchers who provide a methodologically sound proposal, approved by the steering committee to avoid overlap with planned or ongoing analyses. All requests for data can be done via the steering committee, to be contacted via a.mosterd@meandermc.nl. Requests to access the datasets should be directed to a.mosterd@meandermc.nl.
