# Supplementary material for: Colchicine and diabetes in patients with chronic coronary artery disease: insights from the LoDoCo2 randomized controlled trial
Source: Front Cardiovasc Med. 2023 Oct 6;10:1244529. doi: 10.3389/fcvm.2023.1244529 (PMC10587438; doi:10.3389/fcvm.2023.1244529)
Supplement: Supplementary file 1 [file Datasheet1.docx]

***Supplementary Material***

**Colchicine and Diabetes in Patients with Chronic Coronary Artery Disease: Insights From the LoDoCo2 Randomized Controlled Trial**

**Niekbachsh Mohammadnia, Jan Los, Tjerk S.J. Opstal, Aernoud T.L. Fiolet, John W. Eikelboom, Arend Mosterd, Stefan M. Nidorf, Charley A. Budgeon, Jan G.P. Tijssen, Peter L. Thompson, Cees J. Tack, Suat Simsek, Willem A. Bax, Jan H. Cornel^*^, Saloua El Messaoudi**

*** Correspondence:** Jan Hein Cornel, MD, PhD: [**janhein.cornel@radboudumc.nl**](mailto:janhein.cornel@radboudumc.nl)

Supplementary Table S1. Reasons for premature permanent discontinuation of colchicine or placebo in patients with diabetes.

|  | Colchicine (n=492) | Placebo (n=515) |
| --- | --- | --- |
| **Permanent discontinuation of study medication^a^** | | |
| Never received dose, No. (%) | 4 (0.8%) | 4 (0.8%) |
| Had perceived side-effects, No. (%) | 16 (3.3%) | 14 (2.7%) |
| Withdrew from trial^b^, No. (%) | 27 (5.5%) | 22 (4.3%) |
| Were withdrawn by physician or had intercurrent illnesses, No. (%) | 13 (2.6%) | 15 (2.9%) |
| **Total** | 60 (12.2%) | 55 (10.7%) |
| ^a^ Premature permanent discontinuation was determined to have occurred if colchicine or placebo was permanently discontinued more than 30 days before the occurrence of a primary endpoint event, the occurrence of non-cardiovascular death, or the regular end-of-trial date, whichever came first. ^b^ Defined as discontinuation of study medication, not complete withdrawal from trial. | | |

#
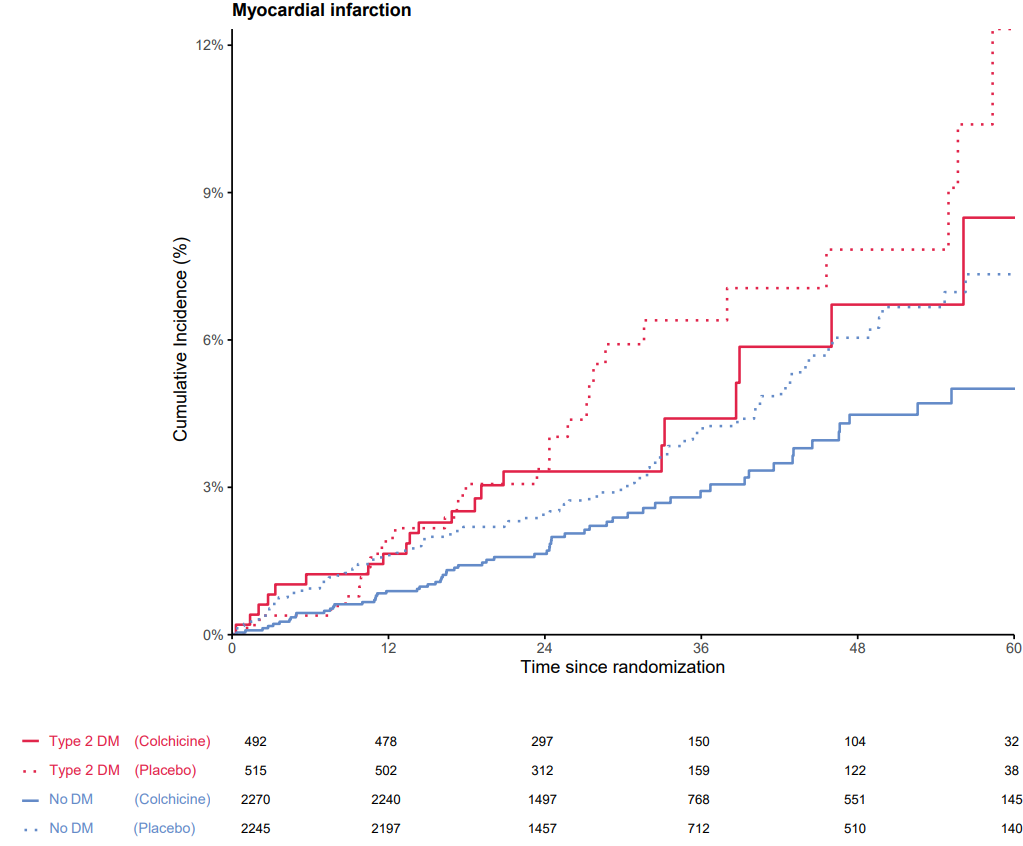


# Supplementary Figure S1. Cumulative incidence of myocardial infarction in the colchicine (solid lines) and placebo (dotted lines) groups in patients with type 2 diabetes at baseline (red lines) and no diabetes (blue lines). The table below the figure shows the numbers at risk at each time point in all groups.

#
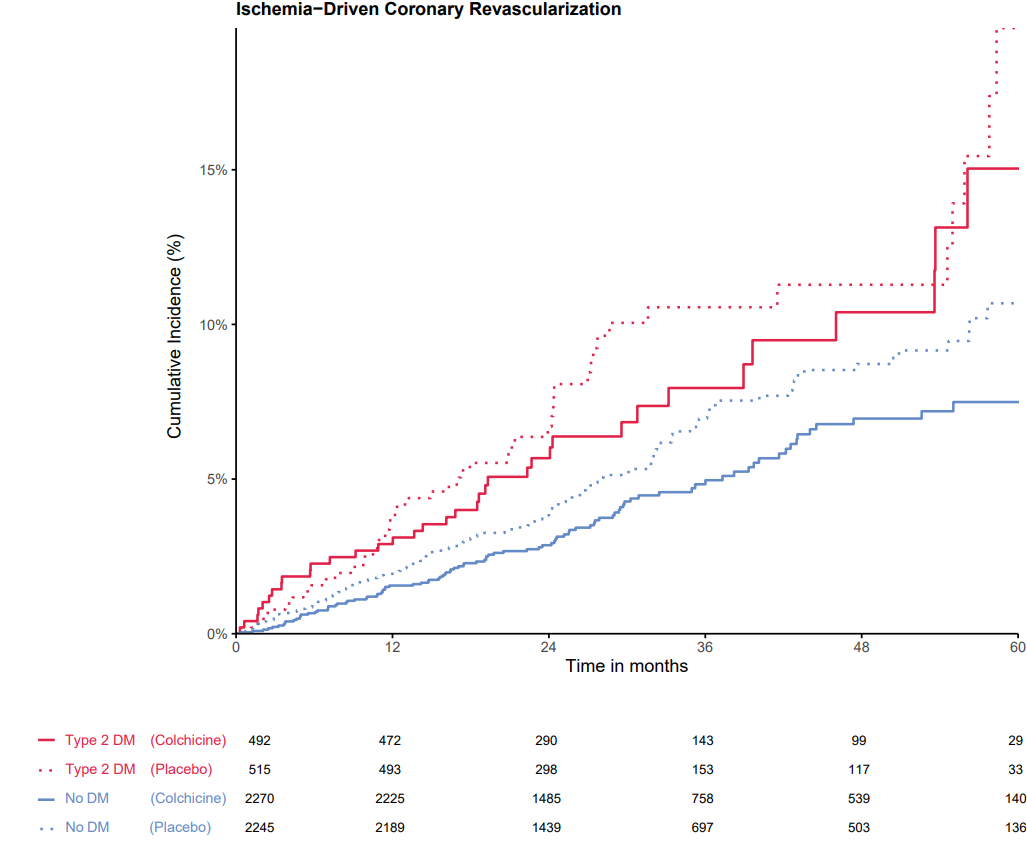


# Supplementary Figure S2. Cumulative incidence of ischemia-driven coronary revascularization in the colchicine (solid lines) and placebo (dotted lines) groups in patients with type 2 diabetes at baseline (red lines) and no diabetes (blue lines). The table below the figure shows the numbers at risk at each time point in all groups.
